# Fifteen-month follow up of an assertive community treatment program for chronic patients with mental illness

**DOI:** 10.1186/s12913-015-1058-y

**Published:** 2015-09-16

**Authors:** Tae-Won Kim, Jong-Hyun Jeong, Young-Hee Kim, Yura Kim, Ho-Jun Seo, Seung-Chul Hong

**Affiliations:** Department of Psychiatry, College of Medicine, The Catholic University of Korea, Seoul, Korea; Suwon Mental Health Center, Suwon, Gyeonggi do Korea; Department of Psychiatry, St.Vincent’s Hospital, College of Medicine, The Catholic University of Korea, 93, Ji-dong, Paldal-gu, Suwon, 442-723 Korea

**Keywords:** Assertive community treatment, ACT, BPRS, Global assessment of functioning, Case management

## Abstract

**Background:**

The aim of this study was to evaluate the effect of an Assertive Community Treatment (ACT) program on psychiatric symptoms, global functioning, life satisfaction, and recovery-promoting relationships among individuals with mental illness.

**Methods:**

Participants were patients at the Suwon Mental Health Center. Thirty-two patients were part of the ACT program and 32 patients matched for age, sex, and mental illness were in a standard case-management program and served as a control group. Follow-up with patients occurred every 3 months during the 15 months after a baseline interview. Participants completed the Brief Psychiatric Rating Scale (BPRS), Global Assessment of Functioning (GAF) Scale, Life Satisfaction Scale, and Recovery-Promoting Relationship Scale (RPRS).

**Results:**

No significant differences were noted in the sociodemographic characteristics of the ACT and the case-management group. According to the BPRS, the ACT group showed a significant reduction in symptom severity, but the ACT program was not significantly more effective at reducing psychiatric symptoms from baseline to the 15-month follow-up compared to the case-management approach. The ACT group showed more significant improvement than the control group in terms of the GAF Scale. Both groups showed no significant differences in the change of life satisfaction and in the change of recovery-promoting relationships. We observed a significant increase in recovery-promoting relationships in the control group, but the degree of change of recovery-promoting relationships through time flow between groups was not significantly different.

**Discussion:**

In this study, we observed that ACT was significantly better at improving the GAF than case management and that participation in ACT was associated with a significant decrease in BPRS scores. However, ACT did not demonstrate an absolute superiority over the standard case-management approach in terms of the BPRS and the measures of life satisfaction and recovery-promoting relationships.

**Conclusions:**

ACT may have some advantages over a standard case management approach.

## Background

Assertive Community Treatment (ACT) has been proposed and implemented as an effective intervention for patients with severe mental illness [1–3]. The core characteristics of ACT are a low patient/therapist ratio, frequent contact with patients in the community, and the availability of crisis intervention 24 h/day [4]. According to the results of randomized controlled trials in the United States, ACT was effective in managing patients with severe mental illness and could reduce the cost of hospital care, promote better treatment outcomes, and lead to increased patient satisfaction [1]. However, other studies performed in United Kingdom did not show significant advantages for ACT and did not replicate the results of studies in the United States [5–8]. The development of standard case-management programs used as a control in the trial and the reduced number of psychiatric wards may have contributed to the discrepancies between studies [9].

In the Republic of Korea, the community support system for patients who are mentally ill is growing and includes global care for diseases and interventions to facilitate functioning. Therefore, it helps patients with mental illness to integrate within the community [10]. Mental health centers play a central role in the community support system, and the case-management program is a core service. The case-management program has been able to decrease symptoms and increase global functioning, but it has had little effect on problem-solving abilities in social situations or recovery of social functioning through the achievement of individual objectives [11]. Thus, the application of an ACT model to the field is needed to facilitate the delivery of more effective services to patients. ACT rests on the following: 1) a team approach to the provision of services to a reasonable number of clients, 2) individualized interventions, 3) a community-based approach, 4) no time limits, and 5) services that are integrated with other community resources [12]. Previous studies have shown that ACT might maintain mental health services, reduce hospitalization, and facilitate the recovery of social functions [13]. Other studies have shown that ACT might be effective in maintaining patients’ employment and housing, which could promote their quality of life and life satisfaction [14, 15].

In Korea, ACT has been evaluated as a regular program for patients since 1990, and the Korean ACT model used over a 6-month period produced significant effects on symptoms, social functioning, and life satisfaction [16]. However, more studies that clearly support the effectiveness of the Korean ACT program are needed. The aim of this study was to evaluate the long-term effects of ACT on the psychiatric symptoms, global functioning, life satisfaction, and recovery-promoting relationships of patients with mental illness during a 15-month follow-up period.

## Methods

### Patients

The subjects in this study were participants in the ACT program at the Suwon Mental Health Center. The inclusion criteria were 1) age 18–60 years; 2) complicated medication, housing, or occupational problems, or legal or familial problems; 3) diagnosis of schizophrenia, bipolar disorder, or major depressive disorder; and 4) agreement to participate in the program and the provision of informed consent from participants and their family members. Participants in the standard case-management program were treated as the control group and were matched with the ACT group in terms of age, sex, diagnosis, and the complexity of their problems in the aforementioned areas to investigate program effectiveness. We provided information about the objectives and process of this study to the participants, and all participants provided written informed consent for this study, which was approved by the institutional review board of St. Vincent’s hospital, the Catholic university of Korea. Baseline interviews were completed in August 2011, and subjects were followed up five times at 3-month intervals for 15 months after the baseline interview. The last interview was performed in November 2012. We treated the 32 ACT participants as the experimental group and the 32 participants in the standard case-management program as the control group.

### Methods

#### Psychiatric symptoms

We used the Brief Psychiatric Rating Scale (BPRS) to evaluate psychiatric symptoms [17]. The BPRS consists of 18 questions, each of which is rated on a seven-point scale (0–6) on which higher scores reflect more severe symptoms. Cronbach’s alpha was set at 0.63–0.88.

#### Global functioning

We used the Global Assessment of Functioning (GAF) Scale to determine patients’ global functioning. This scale has been used to evaluate global functioning since the American Psychiatric Association introduced the Diagnostic and Statistical Manual of Mental Disorders III [18]. Higher GAF scores reflect milder symptoms and lower levels of social and occupational impairment.

#### Life satisfaction

The Life Satisfaction Scale developed by Yang was used to evaluate patients’ satisfaction with their life [19]. This scale covers basic components of satisfaction, such as food, clothing, and shelter, as well as abstract components such as philosophy of life. The scale can evaluate the life satisfaction of the general public and the subjective life satisfaction of patients with mental illness. The scale consists of 33 questions, each of which is rated on a five-point scale on which higher scores reflect higher levels of satisfaction with life. The Cronbach’s alpha was 0.96 when the scale was initially developed.

#### Recovery-promoting relationship

The Recovery-Promoting Relationship Scale (RPRS) developed by Russinova, Rogers, and Ellison was used in this study. This scale asks patients to evaluate the interpersonal skills (eight items) and recovery-promoting strategies (eight items) of their therapist [20]. This 24-item instrument assesses the ability of the therapist to enhance the client’s hopefulness, empowerment, and self-acceptance on a four-point scale. The RPRS has been validated for Koreans with mental illness, and its Cronbach’s alpha was 0.96 [21].

#### ACT case management program

The ACT case-management program included a team leader and a psychiatrist working within a multidisciplinary team approach involving team meetings, registration information, 24 h availability, crisis intervention services, night services, in-field services, individualized service, and family support. Each team consisted of one nurse, one social worker, and a team leader. The psychiatrist was the leader of the whole program. The social worker provided information and helped participants apply for occupational rehabilitation opportunities. The maximum ratio of case managers to patients was 1:5 to increase the intensity, continuity, and consistency of the program. We held a team meeting every morning for case management and treatment planning so we could share and discuss the patients’ information and maintain the program.

An individualized approach was used. A case manager in a designated community identified probable participants, collected patient information, and reported at the team meeting. After discussion, the team decided whether the patient was eligible for registration. After the patient was assigned to a certain team, the case manager and team leader collected more detailed information on the patient, and an individualized treatment plan with individualized objectives was compiled within 1 month. This approach involved continuous case management, monitoring through team meetings, evaluations, and plan revisions as needed.

All services were designed to take place in a patient’s home or community and could occur on a 24 h/day, 365 days/year basis. Patients were able to contact the case-management team via the personnel on night duty and could make phone contact during holidays for purposes of crisis intervention. At the time of ACT program registration, we tried to encourage the participation of family members, patients’ physicians, public organizations, neighbors, and employers. Regular evaluations of the program and efforts to facilitate team core competencies through education and consultations were ongoing. As the case manager completed the scales used in this study, these data were not obtained from independent sources.

#### Case management program

Participants in the standard case-management program were treated as the control group. This program was designed to enhance treatment compliance and provide crisis interventions, in-field services, individualized services, family support, occupational rehabilitation, and financial and legal support. However, these services were not provided by a team; instead, one case manager provided short-term services on an as-needed basis. The case manager-to-patients ratio was 1:45.

### Statistical analysis

Statistical analysis was performed using SPSS 10.0 for Windows (SPSS Inc., Chicago, IL, USA). A *p* value < .05 was considered to indicate statistical significance. Independent-sample *t*-tests, Mann–Whitney *U*-tests, and chi-square tests were used to compare the sociodemographic variables, characteristics of the mental illness, and scores on various scales between the groups. To compare the follow-up data between groups, we used a paired *t*-test, Wilcoxon signed rank-sum test, and repeated-measures analysis of variance.

## Results

### Sociodemographic characteristics

The sociodemographic characteristics of the two groups are presented in Table 1. The mean age of the ACT group was 40.88 ± 10.42 years and that of the control group was 42.03 ± 8.35 years. In terms of education, 22 (68.8 %) members of the ACT group completed at least high school, and 25 (78.1 %) members of the control group completed high school or higher. We found no statistically significant differences in the age, gender, marital status, residence with family, and education between the two groups.

**Table 1:** Sociodemographic and clinical characteristics of patients with mental illness

	ACT (*n* = 32)	Control (*n* = 32)	*P*-value
Age	40.88 ± 10.42	42.03 ± 8.35	0.626
Gender			0.590
Male	21 (65.6 %)	23 (71.9 %)	
Female	11 (34.4 %)	9 (28.1 %)	
Marital status			0.538
Single	21 (65.6 %)	25 (78.1 %)	
Married	3 (9.4 %)	2 (6.3 %)	
Divorced	8 (25.0 %)	5 (15.6 %)	
Residence with family	25 (78.1 %)	29 (90.6 %)	0.168
Education			0.674
6	6 (18.8 %)	3 (9.4 %)	
9	4 (12.5 %)	4 (12.5 %)	
12	16 (50.0 %)	20 (62.5 %)	
> 12 years	6 (18.8 %)	5 (15.6 %)	
Diagnosis			1.000
Schizophrenia	28 (87.5 %)	28 (87.5 %)	
Bipolar disorder	2 (6.3 %)	2 (6.3 %)	
Major depression	2 (6.3 %)	2 (6.3 %)	
Registered disability	26 (81.3 %)	30 (93.3 %)	0.138
Age of first onset			0.426
Teens	8 (25.0 %)	4 (12.5 %)	
Twenties	14 (43.8 %)	18 (56.3 %)	
Thirties	6 (18.8 %)	8 (25.0 %)	
Forties	4 (12.5 %)	2 (6.3 %)	
Occupation before disease onset	25 (78.1 %)	25 (78.1 %)	1.000
Present occupation	6 (18.8 %)	8 (25.0 %)	0.545
BPRS	43.78 ± 12.42	43.41 ± 11.97	0.902
GAF	47.94 ± 12.06	52.41 ± 12.17	0.145
Life satisfaction	95.44 ± 31.35	89.53 ± 27.15	0.424
Recovery-promoting relationships	73.19 ± 18.58	69.59 ± 16.37	0.415

*BPRS* brief psychiatric rating scale, *GAF* global assessment of function

### Characteristics of patients with mental illness

The characteristics of the patients with regard to mental illness are listed in Table 1. In both the ACT and control groups, 28 (87.5 %) patients were diagnosed with schizophrenia, 2 (6.3 %) with bipolar disorder, and 2 (6.3 %) with major depressive disorder. In the ACT group, 26 (81.3 %) patients were registered for disability due to mental illness compared to 30 (93.3 %) in the control group. With respect to age of first onset, 8 (25.0 %) patients in the ACT group experienced the onset of their disease in their teens, and 14 (43.8 %) did so in their twenties. In the control group, 4 (12.6 %) patients experienced disease onset in their teens versus 18 (56.3 %) in their twenties. In both the ACT and control groups, 25 (78.1 %) patients were employed before disease onset. In the ACT group, only 6 (18.8 %) patients were presently employed compared to 8 (25.0 %) in the control group. No significant differences were observed between groups in diagnosis, registered disability, age of first onset, occupation before disease onset, and present occupation.

### Psychiatric symptoms, global function, life satisfaction, and recovery-promoting relationships at baseline

We analyzed psychiatric symptoms, global functioning, life satisfaction, and recovery-promoting relationships at baseline. No significant differences were observed in the BPRS scores between the ACT (43.78 ± 12.42) and control (43.41 ± 11.97) groups. The GAF scores were 47.94 ± 12.06 in the ACT group and 52.41 ± 12.17 in the control group. The life-satisfaction scores in the ACT group were 95.44 ± 31.35, and those in the control group were 89.53 ± 27.15. The score for recovery-promoting relationships in the ACT group was 73.19 ± 18.58, and that in the control group was 69.59 ± 16.37. We observed no statistically significant differences between groups in terms of scores on the BPRS, GAF Scale, Life Satisfaction Scale, and RPRS at baseline (Table 1).

### Changes in psychiatric symptoms

**Fig. 1:**
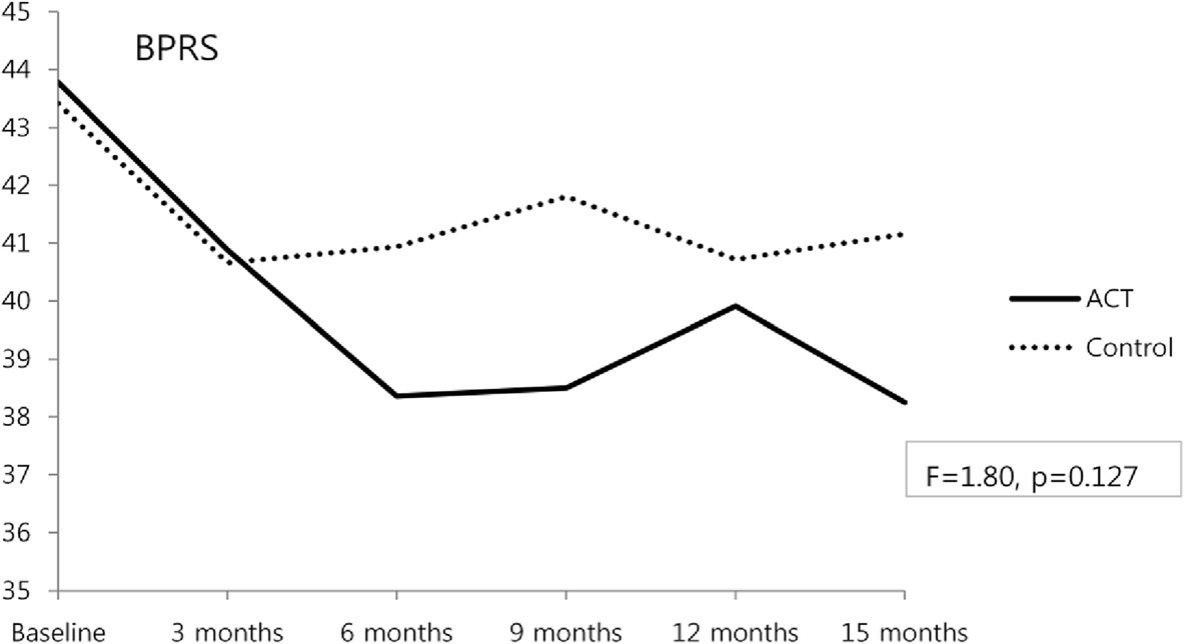
Change in psychiatric symptoms

We administered the BPRS six times at 3-month intervals to determine whether the ACT program was superior to the general case-management program for the reduction of psychiatric symptoms. The BPRS score of the ACT group was 43.78 ± 12.42 at baseline and decreased to 38.25 ± 8.80 at 15 months. In the control group, the BPRS score was 43.41 ± 11.97 at baseline and 41.16 ± 9.81 at 15 months. The reduction in the BPRS score of the ACT group was significant (*p* = .001), but the ACT was not significantly more effective at reducing psychiatric symptoms from baseline to the 15-month follow-up compared to the control group (*F* = 1.80, *p* = .127) (Fig. 1, Table 2).

**Table 2:** Comparisons of psychiatric symptoms, global functioning, life satisfaction scores, and recovery-promoting relationship change between the groups

	Baseline	15 months	Mean difference	*P*-value
BPRS				
ACT (*n* = 32)	43.78 ± 12.42	38.25 ± 8.80	5.53 ± 8.23	0.001*
Control (*n* = 32)	43.41 ± 11.97	41.16 ± 9.81	2.25 ± 9.48	0.189
GAF				
ACT (*n* = 32)	47.94 ± 12.06	52.53 ± 10.57	4.59 ± 9.84	0.013*
Control (*n* = 32)	52.41 ± 12.17	53.25 ± 9.71	0.84 ± 8.19	0.564
Life satisfaction				
ACT (*n* = 32)	95.44 ± 31.35	95.88 ± 24.64	0.44 ± 30.42	0.797
Control (*n* = 32)	89.53 ± 27.15	89.43 ± 26.95	0.94 ± 26.22	0.984
Recovery-promoting relationship				
ACT (*n* = 32)	73.19 ± 18.58	80.56 ± 13.41	7.38 ± 21.19	0.060
Control (*n* = 32)	69.59 ± 16.37	76.47 ± 15.18	6.88 ± 16.26	0.036*

Analyzed by Paired *t*-test and Wilcoxon’s signed rank test

*p < 0.05

### Change in global functioning

**Fig. 2:**
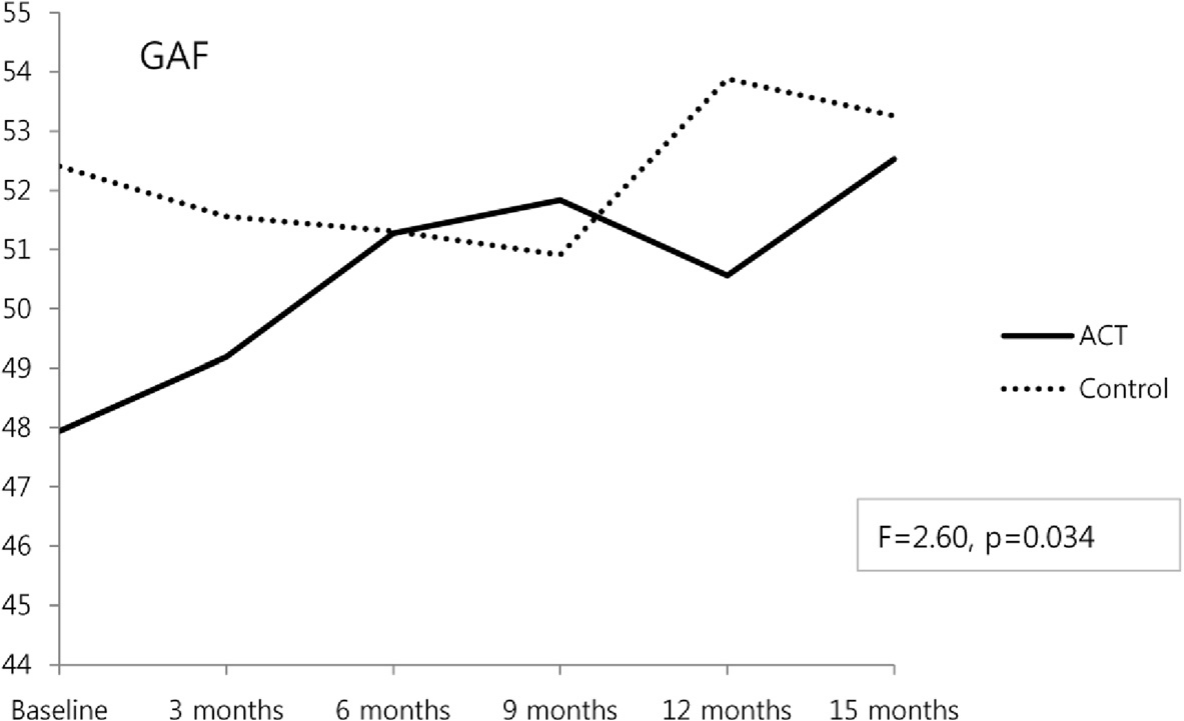
Change in global functioning

We completed the GAF for the two groups six times at 3-month intervals. The GAF scores for the ACT group were 47.94 ± 12.06 at baseline and 52.53 ± 10.57 at 15 months. In the control group, the GAF score was 52.41 ± 12.17 at baseline and 53.25 ± 9.71 at the 15-month follow-up. The increase in the GAF score in the ACT group was significant (*p* = .013), and the ACT group demonstrated a significantly greater increase in global functioning compared to the control group from baseline to the 15-month follow-up (*F* = 2.60, *p* = .034) (Fig. 2, Table 2).

### Change in life satisfaction

We evaluated the life satisfaction of the groups six times at 3-month intervals. In the ACT group, life satisfaction at baseline was 95.44 ± 31.35, changing very little at the 15-month follow-up (95.88 ± 24.64). Life-satisfaction scores in the control group were 89.53 ± 27.15 at baseline and 89.43 ± 26.95 at the 15-month follow-up. Although in the early stages of follow-up, the ACT group showed significant improvement in life satisfaction, the difference in the life satisfaction between the groups from baseline to the 15-month follow-up was not significant (*F* = 0.89, *p* = .490) (Table 2).

### Change in recovery-promoting relationships

We analyzed the recovery-promoting relationships of these two groups six times at 3-month intervals. The RPRS scores for the ACT group were 73.19 ± 18.58 at baseline and 80.56 ± 13.41 at the 15-month follow-up. In the control group, the RPRS score was 69.59 ± 16.37 at baseline, increasing significantly to 76.47 ± 15.18 at the 15-month follow-up (*p* = .036). However, the change in this score between groups from baseline to the 15-month follow-up was not significant (*F* = 1.48, *p* = .210) (Table 2).

## Discussion

In this study, we compared the effect of an ACT program versus that of a case-management approach on the psychiatric symptoms, global functioning, life satisfaction, and recovery-promoting relationships of patients at the Suwon Mental Health Center.

We hypothesized that patients in the ACT group would exhibit greater psychiatric symptom improvement as shown by BPRS scores than the control group receiving case management. The ACT group showed significant reductions in psychiatric symptoms, but these reductions were not significantly greater than those seen in the control group between baseline and the 15-month follow-up. In a previous study, patients who underwent the ACT program showed no significant change in hospitalization period or in symptoms [8]. Another study revealed that the ACT program for disabled patients with mental illness could significantly improve psychiatric symptoms [22]. An ACT program in Japan showed improvement of depressive symptoms and reduction of hospitalization period [23]. In this study, no significant group × time interactions were observed between the two groups in BPRS scores, but the ACT group showed significant decreases in BPRS scores between baseline and 15 months, which suggests that patients treated at a mental health center may have more serious symptoms and require longer follow-up periods to identify the significant effects of intervention types. It may also mean that the contribution of case-management programs to the improvement of patients has been increasing through their continuous development and modification.

The ACT group exhibited a significant increase in global functioning compared to the control group from baseline to the 15-month follow-up. The increase in GAF scores in the ACT group from baseline to 15 months was significant (4.59 ± 9.84, *p* = .013), whereas the control group showed no significant increase (0.84 ± 8.19). In previous Korean studies, patients in the ACT program improved in global functioning [24, 25]. An ACT program may increase patients’ treatment compliance, ability to choose more appropriate solutions for their problems, and ability to adapt to their social and occupational environment, which may increase their integration into the community. Thus, ACT may improve the global functioning of patients.

We expected the ACT program to have a more significant effect on improving patients’ life satisfaction versus the control group, but the difference in life satisfaction between groups from baseline to the 15-month follow-up was not significant. The increase in life satisfaction during the follow-up period in both groups was subtle even though previous studies demonstrated an increase in life satisfaction following the ACT program [26, 27]. Our result is consistent with a Japanese study reporting that the ACT program had no significant relationship with life satisfaction [28]. The Korean ACT program may need revision, and more human and financial resources must be made available to make the ACT program services more intensive. In general, patients with more severe symptoms probably operate under stronger restrictions from ACT team members. These restrictions likely reduce patients’ autonomy, which could then lead to a decrease in their life satisfaction. Therefore, the balance between restriction and autonomy could affect life-satisfaction scores.

The difference between groups in the change in the RPRS scores from baseline to the 15-month follow-up was not significant. However, these scores showed an increasing trend in the ACT group, and they increased significantly from baseline to the 15-month follow-up in the control group. Previous studies showed that ACT team leadership and the case manager’s competence influenced the effect of the ACT program [29]. No previous studies regarding recovery-promoting relationships have been conducted in Korea, and we did not observe a positive effect of the ACT program on recovery-promoting relationships. Future research regarding this topic is needed.

In the previous Korean ACT study, subjects were followed over a 6-month period, and the ACT group showed a significant improvement with regard to psychiatric symptoms, social functioning, and life satisfaction [16]. According to the present study, significant reductions in psychiatric symptoms were observed in the ACT group according to the BPRS and global improvements occurred in functioning as assessed by the GAF. However, scores for life satisfaction and recovery-promoting relationship did not significantly improve in the ACT group. The ACT group showed a significant group × time interaction only with regard to the GAF. This suggests that ACT would have the greatest effect during the earlier part of the program, and that the major role of ACT may involve the improvement of global functioning. We set the maximum ratio of case managers to patients at 1:5 in the ACT group to increase the intensity, continuity, and consistency of the program. However, the case manager-to-patient ratio was 1:45 in the control group. Thus, the provision of more resources and the expenditure of a greater effort by the case manager to the participants in the ACT program may have improved the GAF scores in this study.

The ACT program improved the global functioning of patients with mental illness to a greater extent compared to a standard case-management program. The ACT program also contributed to the relief of psychiatric symptoms. In Korea, hospitalization is preferred to community-based care. However, an ACT program may play an important role in enhancing social inclusion and community integration. Thus, ACT programs should become more common in Korea, and our findings should facilitate this development.

The limitations of this study were as follows: 1) it was performed in one institution with a relatively small number of subjects who were not randomized, which limits the generalizability of the results; 2) a 15-month follow-up period may be too short to identify and detect a lasting change in patients; 3) the ratings were not made by independent raters, which could be a potential source of bias; and 4) the ACT group and control group were not fully matched regarding history of frequent admission, but we tried to match the control group with the ACT group in terms of age, sex, diagnosis, and the complexity of their problems (psychiatric symptoms, degree of disability due to psychiatric problems, global functioning) to reduce the potential bias. Future studies should include more subjects and a longer follow-up period.

## Conclusions

In this study, we found that ACT was significantly more effective in increasing GAF scores than a standard case-management approach and that ACT produced a significant reduction in BPRS scores. However, ACT did not show absolute superiority to a standard case-management approach in terms of BPRS scores, life satisfaction, and recovery-promoting relationships. Nonetheless, ACT may offer several advantages over a standard case-management approach.

## Abbreviations

ACT: Assertive community treatment
BPRS: Brief psychiatric rating scale
GAF: Global assessment of function
RPRS: Recovery-promoting relationship scale
